# Sleep and health-related quality of life in women following a cancer diagnosis: results from the Women’s Wellness after Cancer Program in Australia

**DOI:** 10.1007/s00520-022-07429-0

**Published:** 2022-11-09

**Authors:** Shannon L. Edmed, M. Mamun Huda, Simon S. Smith, Charrlotte Seib, Janine Porter-Steele, Debra Anderson, Alexandra L. McCarthy

**Affiliations:** 1 Institute for Social Science Research, The University of Queensland, 80 Meiers Rd, Indooroopilly, Brisbane, 4068 Australia; 2 ARC Centre of Excellence for Children and Families Over the Life Course, The University of Queensland, Brisbane, Australia; 3 ARC Centre of Excellence for the Digital Child, The University of Queensland, Brisbane, Australia; 4 School of Nursing and Midwifery, Griffith University, Southport Queensland, 4215 Australia; 5 The Wesley Hospital Choices Cancer Support Centre, Brisbane, Australia; 6 School of Nursing, Midwifery and Social Work, The University of Queensland, Brisbane, Australia; 7 Faculty of Health, University of Technology Sydney, New South Wales, Australia; 8 Mater Research Institute, Brisbane, QLD Australia

**Keywords:** Sleep, Cancer, Women’s cancer, Health-related quality of life

## Abstract

**Purpose:**

Sleep disturbance after cancer treatment could compromise recovery. This paper examined the associations between post-treatment sleep problems and health-related quality of life (HRQoL), and the effectiveness of an e-enabled lifestyle intervention on sleep outcomes.

**Methods:**

The Women’s Wellness after Cancer Program (WWACP) was examined in a single blinded, multi-centre randomised controlled trial. Data were collected from 351 women (*M*_*age*_ = 53.2, *SD* = 8.8; intervention *n* = 175, control group *n* = 176) who had completed surgery, chemotherapy and/or radiotherapy for breast, gynaecological or blood cancers within the previous 24 months. Participants completed the Pittsburgh Sleep Quality Index (PSQI) at baseline (prior to intervention randomisation), and at 12 and 24 weeks later. Sociodemographic information, menopausal symptoms (Greene Climacteric Scale) and HRQoL (36-Item Short Form Health Survey; SF-36) were also collected. Linear panel regression was used to examine the association between sleep variables and SF36 Physical Component Summary (PCS) and Mental Component Summary (MCS) scores. A difference-in-difference regression model approach was used to examine the intervention effect on the sleep outcomes.

**Results:**

After adjustment for potential confounders, the sleep variables (except sleep duration) significantly predicted physical, but not mental, HRQoL. There was no statistically significant effect of the intervention on sleep outcomes at 12 or 24 weeks.

**Conclusion:**

Women who have completed treatment for cancer experience sleep problems that are associated with decreased physical HRQoL. Improving sleep through targeted interventions should improve their physical HRQoL. Improved targeting of the sleep components of the WWACP should be explored.

**Supplementary Information:**

The online version contains supplementary material available at 10.1007/s00520-022-07429-0.

## Introduction

As an increasing number of women live with a previous diagnosis of cancer, the importance of supporting the ongoing physical and psychosocial health of this population is an important area of clinical care and research. Women who have completed treatment for cancer experience physical and psychological sequelae that can negatively affect their quality of life (QoL) and health as they recover from treatment [1]. While research in this area is well-established, “recovery” care to help women manage these symptoms is not usual care in practice. As such, there are calls for interventions to improve these symptoms so that women can recover well from their treatment experience [1]. One of the most common persistent health complaints reported by women after cancer treatment is sleep disturbance [1–3]. Poor sleep in women previously treated for cancer has been associated with a range of symptoms and other outcomes including fatigue [4], poorer daytime functioning, mood disturbances, poorer health and diminished health-related QoL (HRQoL) [1, 4].

Sleep is a plausible candidate to explain variability in women’s outcomes, including QoL, after receiving treatment for cancer. Sleep is hypothesised to serve a restorative and recuperative function [5]. Insufficient and disturbed sleep post-cancer treatment could impede recovery, disrupt rehabilitation efforts and compound other psychological, cognitive and health problems, ultimately affecting QoL. Although the association between sleep and HRQoL has been explored in women’s cancer contexts before, much of this research has been conducted in diagnosed women before or during their cancer treatment [6, 7]. Given how common sleep disturbance is after treatment completion [1, 3], more comprehensive research is needed to understand its impact on HRQoL during this stage of the cancer care trajectory. This understanding could inform effective interventions to improve sleep and QoL after cancer treatment.

The years following treatment for cancer are likely a critical period for the development and maintenance of health-improving behaviours that can influence proximal and long-term physical and psychological health outcomes in this population. The Women’s Wellness after Cancer Program (WWACP) was developed to enhance women’s HRQoL after the completion of their breast, blood or gynaecological cancer treatment [8]. The program targets a suite of behaviours, including body composition, physical activity, diet and alcohol consumption via evidence-based health education and health promotion strategies. Among these, strategies for sleep management are also provided. The effectiveness of the program was examined via a multi-centre randomised control trial. Analysis of other trial outcomes of the WWACP intervention (reported elsewhere) found a significant effect of the intervention on HRQoL [9], a significant effect of the intervention for less alcohol intake at 12 weeks, but not at the 24-week follow-up [10], and no significant effect of the intervention for exercise recommendations [11]. The impact of this intervention on sleep has not yet been reported.

The purpose of this paper was to examine the association between sleep problems and HRQoL. We also sought to examine the effectiveness of the WWACP on improving participants’ sleep duration and other sleep problems. It was hypothesised that sleep problems would significantly predict self-reported physical and mental HRQoL, and compared to usual care, women undertaking the WWACP intervention would show greater long-term adherence to sleep recommendations (i.e. sleeping > 7 h per night).

## Methods

### Study design, participants and procedure

The WWACP was a single-blinded, multi-centre randomised controlled trial. Participant recruitment, trial design, measures and procedure have been described in full previously [8]. The protocol was registered with the Australian and New Zealand Clinical Trials Registry: Trial ID: ACTRN12614000800628. In summary, 351 women who had completed chemotherapy (primary or adjuvant) and/or radiotherapy for breast, gynaecological or blood cancers within the previous 24 months were enrolled in this study. Data were collected at baseline (prior to randomisation to the intervention or usual care), 12 weeks (at completion of the intervention) and 24 weeks. In total, 175 (49.9%) participants were randomised to the intervention group and 176 (50.1%) were randomised to the usual care (control) group. Recruitment occurred via five major hospital sites in Queensland, New South Wales, Victoria and Western Australia, and through promotion through partner organisations (e.g. Wesley Hospital Choices Cancer Support Center) and consumer groups (e.g. the National Breast Cancer Foundation’s Register, the Breast Cancer Network of Australia).

Inclusion criteria were age ≥ 18 years, English language proficiency, completion of surgery and primary or adjuvant chemotherapy and/or radiotherapy for breast, blood or gynaecological cancer within the previous 24 months. Access to the internet via a device (mobile phone, laptop or desktop computer) was also required. Participants with metastatic or advanced cancer, inoperable or active locoregional disease or undertaking maintenance chemotherapy for blood cancers were excluded from participation.

Data were collected via online surveys and face to face, depending on the information type. Participants were randomly assigned to either usual care or the WWACP using permuted-block randomisation after completing the baseline data collection assessment. Follow-up data collection occurred at 12 weeks (at completion of the intervention) and 24 weeks (to assess sustained behaviour change) after the baseline assessment.

The WWACP was a 12-week multimodal health education and promotion intervention for women previously treated for cancer that aims to enhance HRQoL. The program was delivered via an e-health-enabled platform, with individual virtual consultations (~ 1 h at weeks 0, 6 and 12) with a cancer nurse occurring for 12 weeks. Participants all received a hard copy journal and or iBook divided into weeks and steps to assist with developing positive lifestyle habits. Topics included managing exercise, nutrition, sleep, stress, menopause and sexuality concerns. There was access throughout the intervention to a website (including podcasts), discussion groups on forums and interactive WWACP iBook. A range of delivery strategies were used to help participants attend to the intervention information, including consultations, motivational coaching and mobile phone text messaging. The nurses worked with participants flexibly to set realistic goals within the participants’ individual physical and psychological capabilities. Fidelity was enhanced by the expert cancer nurses receiving intervention training, including receiving a self-directed protocol manual, and participation in two full days of skills development sessions. Further information about the WWACP intervention content and delivery strategies have been reported previously [8]. The sleep-related content in the program included a section in the book that discussed sleep management strategies and encouraged the use of the six rules for ‘BETTER’ sleep [12]. A podcast discussing sleep approaches was also available for participants. Where participants specifically indicated that improving sleep was a key goal for them, the cancer care nurse would offer support and discussion in this specific area. This might include (but was not limited to) reasons for sleep issues, maintaining a sleep diary, referral back to the general practitioner for ongoing referral to sleep specialists, counselling support and recommendation of other written sleep resources.

This study was approved by Queensland University of Technology Human Research Ethics Committee (Approval No: 1300000335). All participants provided written informed consent.

### Measures

#### Sleep problems

Sleep quality was measured using the Pittsburgh Sleep Quality Index (PSQI) [13]. The PSQI has 19 self-rated items that measure sleep quality and disturbances. For each item, participants were asked to reflect on their usual sleep habits for the previous month. The response formats for each of the items vary, but scoring instructions are used to assign scores from 0 to 3 for each of the 7 subscales or “components”. The 7 component scores represent the following constructs: (1) subjective sleep quality, (2) sleep latency, (3) sleep duration, (4) habitual sleep efficiency, (5) sleep disturbances, (6) use of sleep medication and (7) daytime dysfunction. Scores on these component scores range from 0 to 3, with higher scores indicating more severe difficulty (0 = no difficulty; 3 = severe difficulty). The seven component scores were added together to create one *Global Sleep Quality* score ranging from 0 to 21, with a higher score indicating more severe difficulty across all areas. The additional 5 roommate/bed partner-rated items were not scored in this study. This scale has demonstrated adequate psychometric properties [13], including in cancer patients [4, 14]. The scale is the most commonly used in cancer studies [15]. A cut score of > 5 was used for the *Global Sleep Quality* score to indicate clinically significant sleep problems, consistent with previous recommendations and research [16, 17]. Other sleep outcomes derived from this measure included insufficient sleep duration (< 7 h; i.e. component score > 0); poor sleep quality (“fairly bad” and “very bad” sleep quality; i.e. component score of ≥ 2); poor sleep efficiency (habitual sleep efficiency of “65–74%” and “ < 65%; i.e. component score ≥ 2); frequent sleep disturbance (i.e. “once or twice a week” and “three or more times a week”; i.e. component score ≥ 2).

#### Health-related quality of life

The 36-Item Short Form Health Survey (SF-36) [18, 19] is a widely used non-specific measure of HRQoL. The measure meets high psychometric standards [20, 21]. The 36 items represent eight scales: Physical functioning, role-physical, bodily pain, general health, vitality, social functioning, role-emotional and mental health. These eight scales form two higher order constructs: physical health (the first four scales) and mental health (the latter four scales). These constructs are represented by two summary measures: Physical Component Summary (PCS) measure and Mental Component Summary (MCS) measure. Scoring was completed as per standard instructions, using norm-based scoring algorithms. Lower scores on the MCS reflect “frequent psychological distress, substantial social and role disability due to emotional problems; health in general rated ‘poor’”. Lower scores on the PCS reflect “Substantial limitations in self-care, physical, social, and role activities; severe bodily pain; frequent tiredness; health rated ‘poor’” (p.72) [22].

### Covariates

Sociodemographic and clinical data (e.g. age, education, employment status, marital status, self-reported body mass index (BMI)) were collected via online surveys and face to face, depending on information type. Menopausal symptoms were assessed with the 21-item self-rated Greene Climacteric Scale (GCS) [23], which includes three main scales: psychological (11 items), physical (7 items) and vasomotor (2 items: hot flushes, sweating at night), and single-item “sexual dysfunction” measure that elicits information about “loss of interest in sex”. Pain was measured using the bodily pain subscale of the SF-36. Other data collected included the International Physical Activity Questionnaire Short Form (IPAQ-SF), the Center for Epidemiologic Studies Depression Scale and the Zung Self-rating Anxiety Scale.

### Data analysis

To account for the baseline difference in the sleep outcome prevalence, we used the difference-in-difference regression model approach to examine the intervention effect on the five sleep outcomes. The difference-in-differences estimate is a widely used statistical technique for pre-post intervention control design for efficacy analysis [24, 25]. In this approach, the intervention effect was measured as the difference-in-differences in the proportion of sleep outcomes. The estimate is zero if there is no intervention effect and negative if there is a reduction in the proportion for participants in the intervention group compared to the control participants. The regression model was structured as follows to estimate the intervention effect having adjusted by the difference in the proportion at the baseline:

$$\mathrm{Sleep}\;\mathrm{outcomes}=\mathrm{Intercept}+\mathrm a^\ast\mathrm{Group}+\mathrm b^\ast\mathrm{Time}+\mathrm c^\ast\mathrm{Interaction}+\mathrm{error}$$

The variables were coded as follows: for group, intervention = 1 and control = 0; for time, follow-up (12 or 24 weeks) = 1 and baseline = 0; and interaction = 1 for the measurements of intervention group at follow-up time points, and all others = 0. The c-coefficient is considered as the estimate of the intervention effect. As the sleep outcomes were binary variables, difference-in-differences logistic regression models were used to compare the proportion of sleep outcomes between the intervention and control groups at week 12 and week 24 with reference to their baseline estimates. These models were further extended to the adjusted models by including the predictors of these sleep outcomes, which were identified in another study by Edmed and colleagues’ (submitted). Specifically, the models adjusted for age, education, employment status, marital status, GCS psychological symptoms, GCS vasomotor symptoms, GCS sexual dysfunction and pain.

To examine the association between sleep outcomes and HRQoL (both physical (PCS) and mental (MCS)), a single-exposure-outcome model was used. Due to multicollinearity issues, each of the sleep variables was assessed separately with HRQoL. The outcome variables of PCS and MCS were continuous; with data collected at three time points, we used linear panel regression (*xtreg* command in STATA, panel at participants’ ID level) to examine the association between sleep outcomes and HRQoL after accounting for the dependence due to each participant having repeated observations at three different time points. For adjusted analyses, results were adjusted for covariates identified as predictors of sleep as well as the SF36 PCS and MCS (results not reported), which included age, marital status, education, self-reported body mass index (BMI) and the GCS psychological, vasomotor and sexual dysfunction scales, as identified in another study by Edmed and colleagues (submitted).

### Missing data

There were substantial missing values on the sleep disturbance component score (and consequently the *Global Sleep Quality score*) of the PSQI and for some covariates. As such, we conducted analyses in three different samples: (1) available sample (unadjusted model); (2) complete sample (adjusted model on complete sample after excluding missing values at covariates); (3) imputed full sample (complete sample after imputing missing values). Missing values were imputed using multiple imputations. We used chained equations, a sequence of univariate imputation methods with fully conditional specification of prediction equations using STATA command *mi impute chained*. The imputation routine consisted of 1000 iterations to create 30 imputed data sets. These imputations were then validated. To assess the accuracy of the imputation, several parameters were also examined, such as relative increase in variance, fraction of missing information, degrees of freedom, relative efficiency and the between-imputation and the within-imputation variance estimates. Given that we did not observe any substantial differences between the imputed sample and the complete case sample, we report results from the imputed sample in the main text. The results that report the analyses using complete cases are presented in supplementary Tables 1 and 2.

## Results

Table 1 presents the characteristics of the full sample at the baseline measurement. This table shows that nearly all participants had a previous diagnosis of breast cancer, and most participants were born in Australia. Table 2 shows the distribution of participants’ characteristics across the intervention (*n* = 175) and the treatment as usual (i.e. control; *n* = 176) groups. The between group comparisons show that there were no significant differences for the sample characteristics between the intervention and control groups (Table 2).

**Table 1:** Characteristics of participants at baseline

Characteristic	% (*n*)
Number of cancer-treated women recruited	351
Cancer type; *N* = 284	
- Breast	94.7 (269)
- Other (blood, gynaecological)	5.3 (15)
Mean age (SD)	53.18 (8.77)
Residing state or territory; *N* = 348	
- New South Wales	25.4 (89)
- Victoria	22.2 (78)
- Queensland	25.6 (90)
- South Australia	10.5 (37)
- Western Australia	9.7 (34)
- Tasmania	4. 6 (16)
- Australian Capital Territory	2.0 (7)
Country of birth; *N* = 347	
- Australia	69.7 (242)
- Elsewhere	30.3 (105)
Identifies as Aboriginal and/or Torres Strait Islander; *N* = 345	
- Yes	0.6 (2)
- No	99.4 (343)
Language other than English; *N* = 345	
- Yes	10.4 (36)
- No	89.6 (309)

**Table 2:** Distribution of participant characteristics between intervention and control group at baseline

Covariates	% (*n*)			*P*-value
	Intervention; *N* = 175	Control; *N* = 176	Total; *N* = 351	
Age				
- < 45 year	35.8 (62)	30.9 (54)	33.3 (116)	0.325
- ≥ 45 years	64.2 (111)	69.1 (121)	66. 7 (232)	
- No. of missing	2	1	3	
Country of birth				
- Australia	69.9 (121)	69.5 (121)	69.7 (242)	0.935
- Elsewhere	30.1(52)	30.5 (53)	30.3 (105)	
- No. of missing	2	2	4	
Marital status				
- Married or de facto	22.7 (39)	23.6 (41)	23.1 (80)	0.845
- Else	77.3 (133)	76.4 (133)	76.9 (266)	
- No. of missing	3	2	5	
Employment status				
- Else	13.8 (22)	14.1 (23)	14.0 (45)	0.943
- Employed	86.2 (137)	85.9 (140)	86.0 (277)	
- No. of missing	16	13	29	
Education				
- Low	7.6 (13)	10.3 (18)	9.0 (31)	0.390
- Intermediate	34.3 (59)	32.8 (57)	33.5 (116)	
- High	58.1 (100)	56.9 (99)	57.5 (199)	
- No. of missing	3	2	5	
Income				
- Less than $AU20,000	1.8 (3)	1.8 (3)	1.8 (6)	0.924
- $AU20,000–$80,000	31.7 (52)	29.1 (48)	30.4 (100)	
- Above $AU80,000	66.5 (109)	69.1 (114)	67.8 (223)	
- No. of missing	11	11	22	
Self-reported BMI				
- Else	70.6 (113)	73.5 (122)	72.1 (235)	0.564
- Obese	29.4 (47)	26.5 (44)	27.9 (91)	
- No. of missing	15	10	25	
Physical activity (IPAQ-SF)				
- Low (< 600 MET min/wk)	18.6 (26)	29.0 (40)	23.7 (66)	0.117
- Moderate (600–1199 MET min/wk)	25.0 (35)	22.5 (31)	23.7 (66)	
- High (1200 + MET min/wk)	56.4 (79)	48.6 (67)	52.5 (146)	
- No. of missing	35	38	73	
Menopausal status				
- Pre-menopausal	6.3 (11)	6.9 (12)	6.6 (23)	0.867
- Peri-menopausal	13.8 (24)	12.6 (22)	13.2 (46)	
- Post-menopausal	79.9 (139)	80.6 (141)	80.2 (280)	
- No. of missing	1	1	2	
Greene Climacteric Scale total score				
- 1st tertile	24.5 (35)	21.0 (31)	22.7 (66)	0.586
- 2nd tertile	52.5 (75)	52.7 (78)	52.6 (153)	
- 3rd tertile	23.1 (33)	26.4 (39)	24.7 (72)	
- No. of missing	32	28	60	
Greene Climacteric Scale subscale: psychological				
- 1st tertile (0–5)	34.4 (56)	35.5 (60)	34.9 (116)	0.655
- 2nd tertile (6–9)	36.2 (59)	30.2 (51)	33.1 (110)	
- 3rd tertile (10–28)	29.5 (48)	34.3 (58)	31.9 (106)	
- No. of missing	12	7	19	
Greene Climacteric Scale subscale: vasomotor				
- ≤ 2	53.5 (91)	50.6 (86)	52.1 (177)	0.587
- 3–6	46.5 (79)	49.4 (84)	47.9 (163)	
- No. of missing	5	6	11	
Greene Climacteric subscale: somatic				
- 1st tertile (0–2)	50.3 (83)	42.4 (70)	46.4 (153)	0.131
- 2nd tertile (4–5)	22.4 (37)	23.6 (39)	23.0 (76)	
- 3rd tertile (6–19)	27.3 (45)	33.9 (56)	30.6 (101)	
- No. of missing	10	11	21	
Greene Climacteric subscale: sexual dysfunction				
- No or a little	58.5 (96)	52.4 (88)	55.4 (184)	0.260
- Else	41.5 (68)	47.6 (80)	44.6 (148)	
- No. of missing	11	8	19	
Center for Epidemiologic Studies Depression Scale				
- No depression	70.6 (108)	73.7 (109)	72.1 (217)	0.554
- Yes-clinical depression risk (cut score > 16)	29.4 (45)	26.4 (39)	27.9 (84)	
- No. of missing	22	28	50	
Zung self-rating anxiety score				
- No anxiety	92.0 (137)	92.0 (138)	92.0 (275)	0.986
- Mild to moderate anxiety or greater (cut score > 44)	8.1 (12)	8.0 (12)	8.0 (24)	
- No. of missing	26	26	52	
Bodily Pain scale (SF-36)				
- 1st tertile (0–62)	51.7 (90)	52.0 (91)	51.9 (181)	0.255
- 2nd tertile (63–74)	14.4 (25)	22.3 (39)	18.3 (64)	
- 3rd tertile (75–100)	33.9 (59)	25.7 (45)	29.8 (104)	
- No. of missing	1	1	2	

Percentage is calculated on valid cases (excluding missing cases). *IPAQ-SF*, International Physical Activity Questionnaire - Short Form. SF-36, 36-Item Short Form Health Survey

### Association between sleep and HRQoL

Table 3 shows the association between the sleep outcomes and HRQoL as measured by the SF-36 MCS and PCS. These results show that all the sleep indicators statistically significantly predict physical HRQoL, except for insufficient sleep duration, in both the unadjusted and adjusted analyses. Although the sleep outcomes predict mental HRQoL in the unadjusted analyses, the results were not statistically significant when the results were adjusted for age, marital status, education, self-reported BMI and the GCS psychological, vasomotor and sexual dysfunction scales (Table 3). The statistically significant results of the adjusted analyses show that for women who had sleep problems, the predicted PCS score would be 2.1–3.0 points lower than women who did not have sleep problems, holding all other covariates constant (Table 3).

**Table 3:** Association between sleep and health-related quality of life (HRQoL) of cancer-treated women: regression analysis

	Beta coefficient (95% CI)	
Sleep	Unadjusted	Adjusted*
**PCS**		
- Insufficient sleep duration	− 1.22 (− 2.46, 0.01)	− 0.49 (− 1.75, 0.77)
- Poor sleep quality	− **2.68 (**− **3.95,** − **1.41)**	− **2.14 (**− **3.46,** − **0.81)**
- Poor sleep efficiency	− **2.52 (**− **3.9,** − **1.14)**	− **2.10 (**− **3.53,** − **0.68)**
- Frequent sleep disturbance	− **3.82 (**− **5.13,** − **2.51)**	− **3.02 (**− **4.36,** − **1.67)**
- Clinically significant sleep disturbance	− **3.36 (**− **4.87,** − **1.86)**	− **2.70 (**− **4.30,** − **1.09)**
**MCS**		
- Insufficient sleep duration	− **2.37 (**− **3.79,** − **0.96)**	− 0.49 (− 1.71, 0.73)
- Poor sleep quality	− **4.22 (**− **5.69,** − **2.75)**	− 1.16 (− 2.45, 0.13)
- Poor sleep efficiency	− **3.06 (**− **4.64,** − **1.48)**	− 0.02 (− 1.40, 1.35)
- Frequent sleep disturbance	− **2.82 (**− **4.38,** − **1.27)**	− 0.86 (− 2.23, 0.50)
- Clinically significant sleep disturbance	− **5.69 (**− **7.48,** − **3.89)**	− 1.53 (− 3.10, 0.04)

*Missing imputed analysis. Bolded values indicate statistically significant findings. *MCS*, mental component score; *PCS*, physical component score. Insufficient sleep duration is defined as < 7 h per night (sleep duration component score > 0). Poor sleep quality is defined as PSQI “subjective sleep quality component” score of ≥ 2 (i.e. “fairly bad” and “very bad” sleep quality). Poor sleep efficiency is defined as PSQI “habitual sleep efficiency” score of ≥ 2 (i.e. “65–74%” and “ < 65%”). Frequent sleep disturbance is defined as sleep disturbance component score ≥ 2. Clinically significant sleep disturbance is defined as PSQI *Global Sleep Quality* score > 5

### Effectiveness of the intervention on indicators of sleep disturbance

**Fig. 1:**
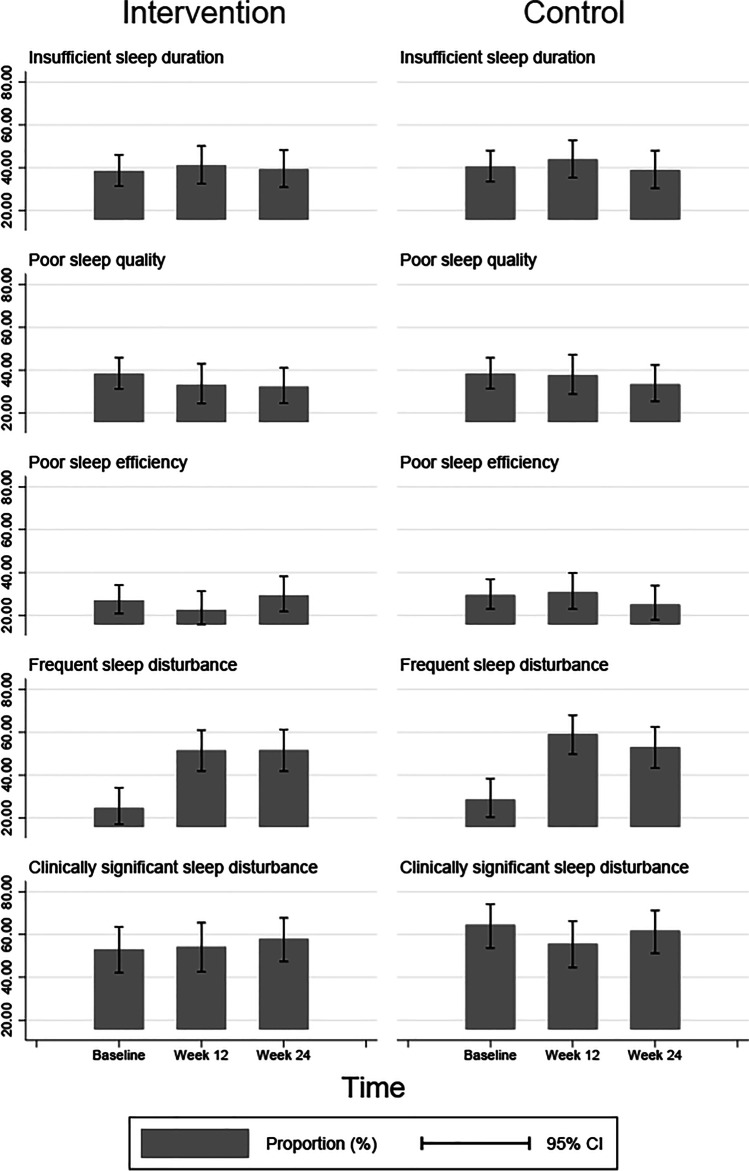
Prevalence of each sleep outcome at baseline, 12 weeks and 24 weeks for Intervention (left) and Control (right) groups

Figure 1 depicts the prevalence of each sleep outcome at baseline, 12 weeks and 24 weeks. The proportion of participants meeting criteria for insufficient sleep duration at baseline is 38% for the intervention group and 40% for the control group. By week 24, 39% of the intervention group and control group meet criteria for insufficient sleep. A similar pattern of results can be observed for other outcomes (Fig. 1 and Table 3). Table 4 reports the unadjusted and adjusted intervention effects at different times. Non-significant trends from the difference-in-difference analysis show that the proportion of sleep problems reduced at 12 weeks, but not at the 24-week follow-up. Overall, there was no statistically significant effect of the intervention on any of the sleep outcomes at any of the follow-up time points.

**Table 4:** Efficacy of the intervention on sleep outcomes among cancer-treated women: difference-in-differences regression analysis

	Proportion in % (95% CI)		Effect* in % (95% CI)	
Outcomes	Intervention	Control	Unadjusted	Adjusted**
Insufficient sleep duration				
- At baseline	38.37 (31.40, 45.86)	40.46 (33.40, 47.95)	–	–
- At week 12	41.03 (32.48, 50.15)	43.80 (35.23, 52.76)	− 0.69 (− 16.93, 15.56)	− 1.69 (− 16.52, 13.14)
- At week 24	39.17 (30.84, 48.17)	38.79 (30.36, 47.95)	2.46 (− 13.70, 18.63)	4.51 (− 10.52, 19.54)
Poor sleep quality				
- At baseline	38.24 (31.23, 45.77)	38.29 (31.37, 45.71)	–	–
- At week 12	32.99 (24.37, 42.93)	37.50 (28.73, 47.17)	− 4.46 (− 21.17, 12.25)	− 4.71 (− 19.00, 9.58)
- At week 24	32.23(24.51, 41.06)	33.33 (25.39, 42.36)	− 1.05 (− 16.78, 14.68)	− 1.30 (− 15.31, 12.7)
Poor sleep efficiency				
- At baseline	26.95 (20.76, 34.19)	29.45 (22.95, 36.90)	–	–
- At week 12	22.52 (15.69, 31.23)	30.70 (22.92, 39.76)	− 5.68 (− 20.72, 9.37)	− 7.39 (− 19.43, 4.65)
- At week 24	29.31 (21.74, 38.23)	25.00 (17.84, 33.84)	6.81 (− 8.26, 21.88)	6.28 (− 5.78, 18.34)
Frequent sleep disturbance				
- At baseline	24.49 (16.98, 33.97)	28.42 (20.26, 38.30)	–	–
- At week 12	51.46 (41.85, 60.96)	59.09 (49.67, 67.89)	− 3.70 (− 21.93, 14.53)	− 7.82 (− 23.9, 8.26)
- At week 24	51.52 (41.72, 61.20)	52.94 (43.25, 62.42)	2.51 (-16.08, 21.09)	-0.48 (-16.6, 15.63)
Clinically significant sleep disturbance				
- At baseline	53.09 (42.22, 63.67)	64.63 (53.72, 74.21)	–	–
- At week 12	54.29 (42.57, 65.55)	55.70 (44.62, 66.24)	− 8.94 (− 24.01, 6.13)	0.88 (− 18.86, 20.62)
- At week 24	57.95 (47.42, 67.81)	61.80 (51.31, 71.29)	− 2.84 (− 17.29, 11.62)	1.56 (− 16.02, 19.14)

*Difference in differences in the proportion of poor sleep outcomes. **Missing imputed analysis. Insufficient sleep duration is defined as < 7 h per night (sleep duration component score > 0). Poor sleep quality is defined as PSQI “subjective sleep quality component” score of ≥ 2 (i.e. “fairly bad” and “very bad” sleep quality). Poor sleep efficiency is defined as PSQI “habitual sleep efficiency” score of ≥ 2 (i.e. “65–74%” and “ < 65%”). Frequent sleep disturbance is defined as sleep disturbance component score ≥ 2. Clinically significant sleep disturbance is defined as PSQI *Global Sleep Quality* score > 5

## Discussion

This paper examined the relationship between sleep problems and HRQoL, and the potential effectiveness of the WWACP intervention. As hypothesised, sleep efficiency, sleep quality, sleep disturbance and clinically significant sleep disturbance significantly predicted physical HRQoL. However, contrary to our hypothesis, these sleep outcomes did not predict mental HRQoL after adjustment for covariates. Also, sleep duration did not predict physical or mental HRQoL after adjustment. Contrary to our hypothesis, participants in the intervention group did not show greater long-term adherence to sleep recommendations (i.e. sleeping > 7 h). Overall, there was no significant effect of the intervention on the sleep duration outcome or any of the other sleep outcomes measured in our study.

We found that all the sleep outcomes except insufficient sleep duration were associated with physical HRQoL, but not mental HRQoL in analyses adjusted for covariates. This may indicate that other factors such as the climacteric-related psychological or vasomotor symptoms contribute more to poorer HRQoL in the mental health domain. Although not explored in this study, there are likely complex pathways between sleep, climacteric symptoms and mental health-related QoL. Further research is needed to understand these complex pathways. Previous studies have also observed a relationship between sleep disturbance and general HRQoL in breast cancer patients at varying stages of the clinical care trajectory [1, 4, 6, 7], including with objectively measured sleep [7]. Our findings contribute to this research by demonstrating the independent association between self-reported sleep problems and physical HRQoL in women treated for cancer using standardised measures of these constructs. The magnitude of this effect was that in women experiencing poor sleep after completing cancer treatment, SF-36 PCS scores were 2.1–3.0 points lower than in women who did not have sleep problems, holding all other covariates constant. As such, unassessed and untreated, sleep problems may undermine physical domains of HRQoL across the cancer care trajectory.

Overall, we found that there was no statistically significant effect of the intervention on any of the sleep outcome measures. There was no support for the a priori hypothesis originally described in the study protocol, which was that compared to usual care, women undertaking the WWACP intervention will show greater long-term adherence to sleep recommendations. The sleep duration recommendations provided by the National Sleep Foundation [26] advise that adults aged between 26 and 65 years should obtain between 7 and 9 h of nightly sleep. In this study, at least 40% of the sample did not meet these recommendations at baseline (38% intervention, 40% control) and there was no statistically significant reduction in the proportion of participants who were exposed to the intervention not meeting these recommendations at 12 weeks (at the end of the intervention) or at 24 weeks (to assess sustained behaviour changed). When interpreting the trends in the effectiveness analysis, the proportion of participants in the intervention group meeting criteria for all poor sleep outcomes reduced at week 12 for many of the outcomes (although not statistically significantly), but this direction of the effect was not sustained at 24 weeks. These trends suggest that any behaviour change or impact of the intervention was not sustained over the longer term.

Although the WWACP included strategies for sleep management, it is likely that the intervention was not effective for this outcome because these strategies were a small component of a larger multimodal lifestyle intervention targeting a broad range of behaviours. In this program, certain behaviours were targeted and worked through intensively when identified as a participant goal. Despite the high prevalence of sleep problems in this sample, women might have prioritised other problems for more intensive focus, which is likely given the array of other symptom concerns in this population (e.g. vasomotor symptoms). Analysis of other trial outcomes of the WWACP intervention (reported elsewhere) did find a significant effect of the intervention on HRQoL at week 12 and sustained at week 24 [9], despite non-sustained improvement of other study outcomes (i.e. alcohol use [10] and exercise recommendations [11]). Overall, although we found no significant effect of the intervention on any of our sleep outcomes, the results from this study can contribute to future meta-analyses. More targeted interventions to improve sleep are needed, where sleep problems are indicated. Further research exploring such interventions, or adaptions to the WWACP are needed.

The results from this study should be interpreted with respect to its limitations. Sleep is a complex construct. Sleep–wake disturbance can be measured subjectively (e.g. self-report) or objectively (e.g. actigraphy, polysomnography). Both ways of measuring sleep provide different, and often complementary information about the nature and impact of the sleep disturbance on the patient. For instance, subjective report provides information about how problematic sleep disturbance is for the patient. However, objective measurement of sleep can overcome limitations associated with self-report, such as recall bias, and reveal potentially important physiological features of sleep. The PSQI includes a range of subscales examining different sleep constructs (e.g. sleep timing, sleep efficiency), but there is lack of specificity by clinically diagnosable disorders. One implication of this lack of diagnostic information is that the experience of “poor sleep” could reflect different sleep disorders that require different treatments. Greater clarity about the nature of sleep disturbance after cancer treatment would be gained if future studies undertook a comprehensive approach to assessing sleep, including the inclusion of objective sleep measurement. We also encountered a significant amount of missing data with this measure on the sleep disturbance component subscale, and as such reported analyses using imputed missing data. The phenomena of obtaining substantial missing data on the PSQI when self-administered has been recognised previously (e.g. 21% of cases lost due to cumulative impact of missing items when calculating the *Global Sleep Quality* score) [14]. Future uses of the PSQI in this population should seek to verify missing participant responses where possible. More broadly, this study utilised a short follow-up period. Longer term monitoring of outcomes might have resulted in different findings. Finally, these results likely do not generalise to women with types of cancers other than breast. Despite these limitations, the study included strong design features of a randomised control trial with repeated follow-up assessments. The primary analyses controlled for the impact of potential confounders. Finally, sleep was assessed using a validated scale. Although previous studies have examined sleep–wake disturbances during recovery from cancer, they have mostly examined sleep–wake disturbance as one of a broader range of symptoms, or commonly as part of QoL measures [27], rather than as a primary outcome with validated scales.

Disturbed sleep after cancer treatment can impede recovery, disrupt rehabilitation efforts and compound other psychological, cognitive and health problems. As a modifiable factor, the treatment of sleep–wake disturbance across the cancer care trajectory can have numerous downstream effects, including improving HRQoL, as suggested by the results of our study. Opportunities to optimise and better target sleep interventions should continue to be explored in this population.

## Supplementary Information

Additional file 1.

Additional file 2.

## Abbreviations

ARC: Australian Research Council
BMI: Body mass index
GCS: Greene Climacteric Scale
HHS: Hospital & Health Service
HRQoL: Health-related quality of life
IPAQ-SF: International Physical Activity Questionnaire - Short Form
M: Mean
MCS: Mental Component Summary
NHMRC: National Health & Medical Research Council
PCS: Physical Component Summary
PSQI: Pittsburgh Sleep Quality Index
QoL: Quality of Life
SD: Standard deviation
SERTA: Study, Education and Research Trust Account
SF-36: 36-Item Short Form Health Survey
WWACP: Women’s Wellness after Cancer Program
