# Supplementary material for: Sleep and health-related quality of life in women following a cancer diagnosis: results from the Women’s Wellness after Cancer Program in Australia
Source: Support Care Cancer. 2022 Nov 9;30(12):10243–53. doi: 10.1007/s00520-022-07429-0 (PMC9715466; doi:10.1007/s00520-022-07429-0)
Supplement: Supplementary file 1 — Additional file 1. [file 520_2022_7429_MOESM1_ESM.docx]

**Supplementary tables**

**Supplementary Table 1: Efficacy of the intervention on sleep related indicators among cancer-treated women: difference in difference regression analysis: complete vs missing imputed analysis**

|  | **Effect* in %; (95% CI)** | |
| --- | --- | --- |
| **Outcomes** | **Adjusted (Complete Case)** | **Adjusted (Missing Imputed)** |
| **Insufficient sleep duration** |  |  |
| - At baseline | -- | -- |
| - At week 12 | -0.80 (-16.24, 14.64) | -1.69 (-16.52, 13.14) |
| - At week 24 | 3.19 (-12.32, 18.70) | 4.51 (-10.52, 19.54) |
| **Poor sleep quality** |  |  |
| - At baseline | -- | -- |
| - At week 12 | -2.93 (-17.46, 11.61) | -4.71 (-19.00, 9.58) |
| - At week 24 | -1.31 (-4.34, 1.71) | -1.30 (-15.31, 12.70) |
| **Poor sleep efficiency** |  |  |
| - At baseline | -- | -- |
| - At week 12 | -7.56 (-19.9, 4.78) | -7.39 (-19.43, 4.65) |
| - At week 24 | 5.46 (-6.4, 17.31) | 6.28 (-5.78, 18.34) |
| **Frequent sleep disturbance** |  |  |
| - At baseline | -- | -- |
| - At week 12 | -6.83 (-24.14, 10.48) | -7.82 (-23.90, 8.26) |
| - At week 24 | 1.41 (-16.05, 18.86) | -0.48 (-16.60, 15.63) |
| **Clinically significant sleep disturbance** |  |  |
| - At baseline | -- | -- |
| - At week 12 | -2.28 (-22.17, 17.61) | 0.88 (-18.86, 20.62) |
| - At week 24 | -2.83 (-20.93, 15.27) | 1.56 (-16.02, 19.14) |

*Difference in differences in the proportion of poor sleep outcomes
