# Supplementary material for: Sleep and health-related quality of life in women following a cancer diagnosis: results from the Women’s Wellness after Cancer Program in Australia
Source: Support Care Cancer. 2022 Nov 9;30(12):10243–53. doi: 10.1007/s00520-022-07429-0 (PMC9715466; doi:10.1007/s00520-022-07429-0)
Supplement: Supplementary file 2 — Additional file 2. [file 520_2022_7429_MOESM2_ESM.docx]

**Supplementary Table 2: Association between sleep and health-related quality of life (HRQoL) of cancer-treated women: complete vs missing imputed analysis**

|  | **Beta Coefficient, (95% CI)** | |
| --- | --- | --- |
| **Sleep** | **Adjusted (Complete Case)** | **Adjusted (Missing Imputed)** |
| **PCS** |  |  |
| - Insufficient sleep duration | -0.77 (-2.19, 0.66) | -0.49 (-1.75, 0.77) |
| - Poor sleep quality | -2.37 (-3.85, -0.9) | -2.14 (-3.46, -0.81) |
| - Poor sleep efficiency | -2.33 (-3.95, -0.72) | -2.1 (-3.53, -0.68) |
| - Frequent sleep disturbance | -2.54 (-4.04, -1.05) | -3.02 (-4.36, -1.67) |
| - Clinically Significant sleep disturbance | -2.89 (-4.65, -1.13) | -2.7 (-4.3, -1.09) |
| **MCS** |  |  |
| - Insufficient sleep duration | -0.32 (-1.68, 1.03) | -0.49 (-1.71, 0.73) |
| - Poor sleep quality | -0.94 (-2.36, 0.49) | -1.16 (-2.45, 0.13) |
| - Poor sleep efficiency | 0.04 (-1.48, 1.57) | -0.02 (-1.40, 1.35) |
| - Frequent sleep disturbance | -1.05 (-2.54, 0.44) | -0.86 (-2.23, 0.50) |
| - Clinically Significant sleep disturbance | -1.27 (-2.99, 0.45) | -1.53 (-3.10, 0.04) |
